# Peak Width of Skeletonized Mean Diffusivity as a Marker of Diffuse Cerebrovascular Damage

**DOI:** 10.3389/fnins.2020.00238

**Published:** 2020-03-19

**Authors:** Audrey Low, Elijah Mak, James D. Stefaniak, Maura Malpetti, Nicolas Nicastro, George Savulich, Leonidas Chouliaras, Hugh S. Markus, James B. Rowe, John T. O’Brien

**Affiliations:** ^1^Department of Psychiatry, University of Cambridge, Cambridge, United Kingdom; ^2^Division of Neuroscience and Experimental Psychology, University of Manchester, Manchester, United Kingdom; ^3^Department of Clinical Neurosciences, University of Cambridge, Cambridge, United Kingdom; ^4^Division of Neurology, Department of Clinical Neurosciences, Geneva University Hospitals, Geneva, Switzerland

**Keywords:** small vessel disease, magnetic resonance imaging, diffusion tensor imaging, white matter hyperintensities, cognition, dementia

## Abstract

**Background:**

The peak width of skeletonized mean diffusivity (PSMD) has been proposed as a fully automated imaging marker of relevance to cerebral small vessel disease (SVD). We assessed PSMD in relation to conventional SVD markers, global measures of neurodegeneration, and cognition.

**Methods:**

145 participants underwent 3T brain MRI and cognitive assessment. 112 were patients with mild cognitive impairment, Alzheimer’s disease, progressive supranuclear palsy, dementia with Lewy bodies, or frontotemporal dementia. PSMD, SVD burden [white matter hyperintensities (WMH), enlarged perivascular spaces (EPVS), microbleeds, lacunes], average mean diffusivity (MD), gray matter (GM), white matter (WM), and total intracranial volume were quantified. Robust linear regression was conducted to examine associations between variables. Dominance analysis assessed the relative importance of markers in predicting various outcomes. Regional analyses examined spatial overlap between PSMD and WMH.

**Results:**

PSMD was associated with global and regional SVD measures, especially WMH and microbleeds. Dominance analysis demonstrated that among SVD markers, WMH was the strongest predictor of PSMD. Furthermore, PSMD was more closely associated to WMH than with GM and WM volumes. PSMD was associated with WMH across all regions, and correlations were not significantly stronger in corresponding regions (e.g., frontal PSMD and frontal WMH) compared to non-corresponding regions. PSMD outperformed all four conventional SVD markers and MD in predicting cognition, but was comparable to GM and WM volumes.

**Discussion:**

PSMD was robustly associated with established SVD markers. This new measure appears to be a marker of diffuse brain injury, largely due to vascular pathology, and may be a useful and convenient metric of overall cerebrovascular burden.

## Introduction

The clinical significance of cerebral small vessel disease (SVD) in cognitive impairment, dementia, and stroke creates an urgent need to identify and develop valid markers of relevance to SVD (Pantoni, 2010). Due to difficulties in detecting perturbations to the small vessels of the brain, SVD is traditionally measured via parenchymal alterations visible on magnetic resonance imaging (MRI). Specifically, four core MRI features have been identified as markers of SVD (Huijts et al., 2013; Wardlaw et al., 2013a, b; Staals et al., 2014, 2015), namely white matter hyperintensities (WMHs), enlarged perivascular spaces (EPVS), microbleeds, and lacunes. Although widely accepted as markers of SVD, lesions like WMH can have mixed etiologies linked to inflammation, Wallerian degeneration, and degenerative (tau) pathology (McAleese et al., 2015, 2017; Low et al., 2019).

However, due to the generally weak associations of these SVD markers with clinical features such as cognitive impairment (Nitkunan et al., 2008; Patel and Markus, 2011; van der Holst et al., 2017), coupled with the labor-intensity of quantifying these markers, there is a need to identify and validate proxy markers of SVD that can be applied to very large studies.

Diffusion tensor imaging (DTI) has been increasingly suggested as a potential marker of cerebrovascular damage, given its sensitivity to microstructural alterations (Croall et al., 2017). Recently, a new fully automated measure, known as the peak width of skeletonized mean diffusivity (PSMD), has been proposed as a marker of SVD (Baykara et al., 2016). PSMD is a DTI-derived measure based on skeletonization and histogram analysis and is calculated as the difference between the 95th and 5th percentile of mean diffusivity (MD) values within the masked MD skeleton. The use of skeletonized maps of MD eliminates contamination from cerebrospinal fluid (CSF), and the histogram-based approach enhances the ability to characterize subtle, diffuse diseases in the brain, such as SVD. Promising results have emerged since its introduction. Importantly, PSMD outperformed established MD parameters in terms of association with processing speed, including mean, median, peak height, full width at half maximum (Baykara et al., 2016). On top of processing speed (Baykara et al., 2016; Deary et al., 2018), PSMD is also strongly related to global cognition and executive functions (Wei et al., 2019). Furthermore, PSMD has been shown to be closely associated with higher WMH lesion load (Wei et al., 2019), and was higher in patients with multiple sclerosis and CADASIL compared to healthy adults (Vinciguerra et al., 2019).

To further assess PSMD as a possible marker of SVD, our first broad aim was to examine its relevance to vascular pathology by investigating (1) its association with each of the four established markers of SVD (WMH, EPVS, microbleeds, lacunes), (2) whether it was more strongly influenced by vascular markers compared to markers of general neurodegeneration, and (3) the spatial overlap of PSMD and WMH severity. The second broad aim of our study was to establish the clinical relevance of PSMD by examining (1) the relationship between PSMD and cognition, and (2) the relative importance of PSMD to cognition, compared to traditional SVD markers. We hypothesized that PSMD would be significantly related to the four markers of SVD and cognition. Topographically, we expected that regions with greater WMH would reflect heightened PSMD scores.

## Materials and Methods

A total of 145 participants were recruited as part of the Neuroinflammation in Memory and Related Other Disorders (NIMROD) study, a multimodal cohort study comprising of older adults with varying degrees of cognitive impairment and dementia subtypes (Bevan-Jones et al., 2017). Briefly, cognitively impaired participants were recruited through memory clinics in and around Cambridgeshire, or via the Dementias and Neurodegeneration specialty of the Clinical Research Network (DeNDRoN), the Join Dementia Research (JDR) platform^1^, while cognitively healthy participants were recruited from DeNDRON, JDR, and among friends, partners, and spouses of patients. Participants with major psychiatric disorders, MRI contraindications, claustrophobia, previous head injury, or systemic inflammatory disease were excluded from the study. A majority of participants had some degree of cognitive impairment (*n* = 112), including mild cognitive impairment (*n* = 20), Alzheimer’s disease (*n* = 16), progressive supranuclear palsy (*n* = 23), dementia with Lewy bodies (*n* = 26), late life depression (*n* = 6), and frontotemporal dementia (*n* = 21) (for diagnostic criteria, see Bevan-Jones et al., 2017). The remainder (*n* = 33) were cognitively healthy, i.e., Mini-Mental State Examination (MMSE) > 26, with an absence of regular memory symptoms, symptoms suggestive of dementia, or unstable or significant medical illness. Participants underwent clinical and cognitive assessments, and MRI scans. Cognition was assessed using the MMSE and Addenbrooke’s Cognitive Examination-Revised (ACE-R). Detailed procedures have been previously described (Bevan-Jones et al., 2017).

### Image Acquisition

Participants underwent MRI scanning at the Wolfson Brain Imaging Centre (WBIC) on a Siemens 3T Tim Trio or Verio (Siemens Healthcare, Erlangen, Germany). Three-dimensional T1-weighted sequences [176 slices, 1.0-mm thickness, repetition time (TR) = 2300 ms, echo time (TE) = 2.98 ms, field of view = 256 × 240 mm^2^, flip angle = 9°, voxel size = 1 × 1 × 1mm^3^], T2 fluid attenuated inversion recovery (FLAIR) images (75 slices, 2.0 mm thickness, TR = 12,540 ms, TE = 132 ms, flip angle = 120°), susceptibility-weighted imaging (SWI) (40 slices, 2.0 mm thickness, TR = 35 ms, TE = 20 ms, flip angle = 17°, acquisition matrix 256 × 240; voxel size = 1 × 1 × 2mm^3^), and diffusion-weighted imaging (63 slices, 2.0 mm thickness, 63 diffusion directions, TR = 11,700 ms, TE = 106 ms, *b*-value 1 = 0 s/mm^2^, *b*-value 2 = 1000 s/mm^2^, acquisition matrix 96 × 96; voxel size = 2 × 2 × 2mm^3^) were obtained.

### Peak Width of Skeletonized Mean Diffusivity

**FIGURE 1:**
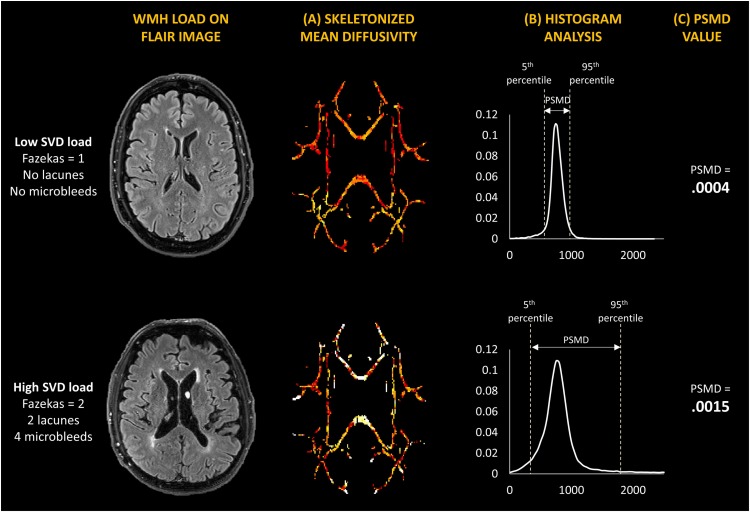
Illustration of **(A)** white matter hyperintensity load on MRI, **(B)** skeletonized mean diffusivity maps, and **(C)** histogram analysis used to obtain PSMD value. Two study participants are compared – one with low SVD burden (Top) and one with high SVD burden (Bottom).

Peak width of skeletonized mean diffusivity was computed automatically using a modified version of the automated procedure described by Baykara et al. (2016). The shell script is freely available online^2^. However, instead of performing eddy current corrections using the default *eddy_correct* tool, we modified the script to use the *eddy* tool which performs outlier replacement in areas of partial or complete signal dropout. Brain masks were created with *Brain Extraction Tool* (*BET*) in FSL^3^ (FMRIB, Oxford, United Kingdom), and diffusion tensor and scalar diffusion parameters were estimated using *DTIFIT*. Computation of PSMD involved two steps: (1) the skeletonization of MD data and (2) histogram analysis. The Tract-based Spatial Statistics (TBSS) pipeline on FSL was used to skeletonize DTI data, i.e., creating a common white matter (WM) skeleton, starting by registering all fractional anisotropy (FA) volumes to standard space using the non-linear registration tool FNIRT and the standard space FMRIB 1 mm FA template. A WM skeleton is then created from registered FA volumes at a threshold FA value of 0.2, and MD volumes are projected onto the FA skeleton. This MD skeleton is further masked with a template skeleton thresholded at an FA value of 0.2 to avoid CSF contamination. Using histogram analysis, PSMD is calculated as the difference between the 95th and 5th percentile of the voxel-based MD values within the masked MD skeleton (Figure 1). Mean and standard deviations of FA and MD were also obtained from the same skeletonized maps in FSL. All study samples were processed using the same pipeline, template skeleton, and mask. Quality assessment was conducted using the *eddy_quad* tool in FSL, and through visual inspection of all images. To examine regional associations, we further computed region-specific PSMD values. In order to compare regional PSMD directly with regional WMH volumes, we extracted lobar regions from skeletonized MD images by applying the same lobar mask used in WMH segmentation registered to the same standard space, and conducted separate histogram analyses on each region to extract PSMD values from each lobe.

Head motion of each subject was obtained using *eddy_quad* on FSL. To account for individual differences in head movement contributing to diffusion indices, head motion was included as a covariate in all analyses involving PSMD.

### Quantification of SVD

#### Semi-Quantitative Measurements

White matter hyperintensities were rated visually by experienced raters, blinded to all clinical information on FLAIR images according to the Fazekas scale (Fazekas et al., 1987). Within each hemisphere, periventricular (PVH) and deep WMH (DWMH) were rated separately on the following scale: PVH (0 = absent; 1 = “caps” or pencil-thin lining; 2 = smooth “halo”; 3 = irregular PVH signal extending into the deep WM), DWMH (0 = absent; 1 = punctate foci; 2 = beginning confluence; 3 = large confluent areas). All scans were manually rated by a single rater (AL), and a randomly selected subset of one-third was rated by a second rater (JS) for reliability testing. The intra-class correlation coefficient (ICC) was 0.85. Twenty percent of all scans were randomly selected and re-rated by the first rater (AL) to assess intra-rater reliability (ICC 0.89). EPVS were rated on T2-weighted images using a validated visual rating scale (Potter et al., 2015). EPVS were rated separately in the basal ganglia (BG), centrum semiovale (CSO), and midbrain, as EPVS in different regions have been suggested to have different underlying pathologies. As per the validated scale, EPVS-BG and EPVS-CSO were rated on a 5-point scale ranging from 0 to 4, accordingly: 0 (none), 1 (1–10), 2 (11–20), 3 (21–40), and 4 (>40). EPVS in the midbrain were rated dichotomously based on the presence or absence of EPVS. Cerebral microbleeds were identified on SWI using the Microbleed Anatomical Rating Scale (MARS) (Gregoire et al., 2009). Following the recommendations made by Greenberg et al. (2009), microbleeds were defined as areas of round/ovoid black signals, excluding tubular or linear structures (Greenberg et al., 2009). Suspected microbleeds were cross-validated on T1- and T2-weighted scans to exclude microbleed “mimics.” In instances of uncertainty, microbleeds were labeled as “possible microbleeds” – this includes situations whereby microbleeds cannot be distinguished from vascular flow voids. Such cases of “possible microbleeds” were excluded from analysis, and only “definite microbleeds” were analyzed. Three participants did not undergo SWI scans and therefore did not have data on microbleeds. Lacunes were identified using T1-weighted, T2-weighted, and FLAIR images, following the STandards for ReportIng Vascular changes on nEuroimaging (STRIVE) guidelines (Wardlaw et al., 2013a, b). Size thresholds for lacune classifications were set at a minimum of 3 mm in diameter (to avoid misclassifications of EPVS as lacunes), and a maximum of 15 mm (to exclude lesions resulting from non-SVD etiologies). Lacunes and microbleeds were classified according to location as deep (e.g., BG, thalamus, internal capsule) or lobar (e.g., CSO) lesions.

#### Quantitative Measurements

White matter hyperintensity volumes were obtained using an automated script on the Statistical Parametric Mapping 8 (SPM8) suite^4^ ; details on the procedures involved have been described previously (Firbank et al., 2003; Smart et al., 2011). Briefly, SPM8 was used to perform segmentation of T1-weighted images into GM, WM, and CSF, based on prior probability maps. Using the GM and WM maps, a brain mask was created and used to perform removal of non-brain matter from the FLAIR images. WMH maps were obtained using threshold-based segmentation at a threshold of 1.40 times the modal pixel intensity, i.e., lesions with pixel intensity more than 1.40 times the modal intensity were included in the WMH map. WMH were segmented into PVH and DWMH, and regionally by lobar regions (frontal, parietal, occipital, temporal) as previously described (Firbank et al., 2003). All generated segmentations were manually reviewed by experts (NN, AL, and EM) and compared against raw FLAIR images to identify misclassifications. Misclassifications were manually corrected, and consensus was met among observers in cases of uncertainty. Sixteen participants did not have data on WMH volumes due to missing volumetric FLAIR images or failed quality assessment, and were excluded from analyses involving WMH volumes. Total SVD burden was computed using a point-based system based on the presence or absence of each of the four SVD markers, according to cut-offs defined by Staals et al. (2014).

White matter hyperintensities, GM, and WM volumes were normalized by TIV to account for individual differences in head size. As WMH volumes were right-skewed, square-root transformations were performed on normalized WMH volumes.

### Variables Included in Analyses

For each individual, PSMD value, GM volume, WM volume, mean MD, TIV, SVD, and cognitive data were available. Regional data for each SVD marker were as such – (1) WMH: PVH and DWMH, (2) EPVS: CSO, BG, and midbrain, (3) microbleeds: deep and lobar, and (4) lacunes: deep and lobar. Global measures of each SVD type were defined as: (1) total WMH volume normalized for TIV, (2) EPVS-BG, (3) total number of microbleeds, (4) total number of lacunes, and (5) total SVD burden score (Staals et al., 2014). Lacking an established whole-brain measure of EPVS, EPVS-BG were selected to represent “global” EPVS, given its relevance in vascular pathology such as hypertensive arteriopathy and WMH, as opposed to EPVS-CSO which are more commonly linked to cerebral amyloid angiopathy (CAA) (Charidimou et al., 2017). Measures of cognition were MMSE and ACE-R scores. Covariates were gender, age, diagnosis (healthy control, mild cognitive impairment, Alzheimer’s disease, progressive supranuclear palsy, dementia with Lewy bodies, and frontotemporal dementia) and head motion during scanning.

### Statistical Analysis

Statistical analysis was performed using R and SPSS V.21.0. Normality of continuous data was tested using the Shapiro–Wilk test. Parametric data were analyzed using either independent *t*-tests or analysis of variance (ANOVA), while non-parametric data were analyzed using the Wilcoxon rank-sum test or Kruskal–Wallis test. Chi-square tests of independence were used for group comparisons of categorical variables. Robust linear regression modeling was performed to examine associations between PSMD, SVD, and cognition (i.e., ACE-R score). Dominance analysis was conducted to assess the relative importance of variables in explaining an outcome. Dominance analysis improves on earlier methods of assessing relative importance (e.g., standardized regression coefficients, squared beta weights, squared zero order correlations) due to its ability to account for correlations between predictors in multivariate analysis (Johnson and Lebreton, 2004). This is an important feature for the purposes of our study, given the close associations between various brain measures.

The specific statistical tests conducted for each analyses are as follows: Robust correlation analysis was conducted to assess the influence of head motion on PSMD and MD. Fisher’s *r*-to-*z* transformation was used to statistically test the difference between correlation coefficients. Robust linear regression modeling was performed to examine associations between PSMD and individual SVD markers (global and regional). For each SVD marker, two models were estimated. The first model adjusted only for head motion, while the second model adjusted for head motion, gender, age, and diagnostic group. The relative contributions of each global SVD marker to PSMD were examined using dominance analysis. Associations between regional PSMD and regional WMH volumes were analyzed using robust correlational analysis. Relative weights analysis and dominance analysis were conducted in R to test the significance of regional differences in each SVD type (e.g., lobar microbleeds vs. deep microbleeds), and the relative importance of PSMD and SVD markers to ACE-R score. Dominance analysis was also conducted to compare the relative contribution of WMH, GM, and WM volumes to PSMD. Diagnostic group and ACE-R scores were further added in the model as covariates to control for the cause and degree of cognitive impairment. Receiver operating characteristic (ROC) curve analysis was conducted to examine the ability of PSMD and SVD markers to distinguish cognitively impaired from cognitively healthy participants. Area under the curve (AUC) of ROC curves were compared using Delong’s test. To correct for multiple comparisons, Holm–Bonferroni corrections were conducted. Statistical significance was set at *p* < 0.05.

## Results

Sample characteristics, cognitive scores, and brain imaging features are presented in Table 1. The sample was majority male (60.7%), had a mean age of 70.6 years (SD 7.8), and an average of 13.3 years of education (SD 2.9). Participants had a mean MMSE score of 26.4 and mean ACE-R score of 79.4. In terms of cerebrovascular burden, 37% had significant WMH, defined as PVH extending into the deep WM (Fazekas 3) and/or confluent or early confluent DWMH (Fazekas 2–3). 45% displayed moderate to severe EPVS in the BG, 35% possessed one or more lacune, and 35% had at least one microbleed. The sample had a mean total SVD burden score (Staals et al., 2014) of 1.50 (out of 4), with 19% scoring a 3 or 4.

**TABLE 1:** Participant characteristics.

*n*	145
**Demographics**	
Sex, % female	39.3
Age (years)	70.6 (7.8)
Education (years)	13.3 (2.9)
**Cognition**	
MMSE	26.4 (3.8)
ACE-R	79.4 (15.0)
**Vascular risk**	
Hypertension (%)	25.8
Hyperlipidemia (%)	27.5
Diabetes Mellitus (%)	6.1
Current smoker (%)	6.4
**MRI features**	
GM volume^1^	39.0 (2.9)
WM volume^1^	32.3 (2.7)
Mean MD	0.77 (0.04)
Mean FA	0.49 (0.03)
WMH (Fazekas scale; 0–3)	1.83 (0.83)
WMH (volume)^1^	0.69 (0.71)
EPVS (basal ganglia; 0–4)	1.51 (0.63)
Microbleeds (count)	1.44 (6.3)
Lacunes (count)	0.86 (2.7)
Total SVD burden score (0–4)	1.50 (1.12)
PSMD	5.51*e*−4 (1.03*e*−4)

*Data are presented as mean (standard deviation), with exception of gender. ^1^Normalized volume adjusting for total intracranial volume. Only n = 129 had data on WMH volume, and n = 142 had data on microbleeds, n = 132 had data on hypertension, n = 131 had data on hyperlipidemia and diabetes, n = 140 had data on smoking. GM, gray matter; WM, white matter; MD, mean diffusivity; WMH, white matter hyperintensities; EPVS, enlarged perivascular spaces; SVD, small vessel disease; PSMD, peak width of skeletonized mean diffusivity.*

In robust correlation analysis, PSMD values were significantly influenced by the degree of head motion during scanning (*r* = 0.265, *p* = 0.001), highlighting the importance of adjusting for head motion in analyses involving PSMD. Mean MD was also influenced by head motion (*r* = 0.186, *p* = 0.026). Fisher’s *r*-to-*z* transformation showed that the extent to which PSMD and MD were influenced by head motion were not statistically different (*z* = −1.19, *p* = 0.233).

### Global Associations Between PSMD and SVD Markers

Robust linear regression models were used to examine associations between PSMD and SVD markers in separate models for each marker. Controlling only for head motion, almost all global and regional SVD markers and total SVD burden score were significantly associated with PSMD (*t* = 3.03–8.02, *p* ≤ 0.001–0.003), with exception of midbrain EPVS and deep lacunes (Table 2). Accounting for multiple comparisons using Holm–Bonferroni correction, WMH, microbleeds, EPVS in the CSO and BG, lobar lacunes, overall lacune load, and total SVD burden score remained significantly associated with PSMD. However, upon controlling for gender, age, and diagnosis, in addition to head motion, only associations with WMH, microbleeds, and lobar lacunes remained significant (*t* = 2.14–5.08, *p* ≤ 0.001–0.038) (Table 2).

**TABLE 2:** Association between PSMD and markers of SVD using robust linear regression.

	Model 1		Model 2	
	*t*	*p*	*t*	*P*
**WMH (Fazekas score)**				
Periventricular	5.67	<0.001***^†^	3.92	<0.001***^†^
Deep	3.79	<0.001***^†^	2.14	0.038*
Overall	5.73	<0.001***^†^	3.87	<0.001***^†^
**WMH volume**				
Periventricular	7.70	<0.001***^†^	4.71	<0.001***^†^
Deep	5.49	<0.001***^†^	2.99	0.007**
Overall	8.02	<0.001***^†^	5.08	<0.001***^†^
**EPVS**				
Centrum semiovale	3.09	0.002**^†^	0.71	0.476
Basal ganglia	3.41	<0.001***^†^	1.46	0.147
Midbrain	0.30	0.761	−1.56	0.114
**Microbleeds (count)**				
Lobar	4.15	<0.001***^†^	4.85	<0.001***^†^
Deep	3.58	<0.001***^†^	4.10	<0.001***^†^
Total number	4.14	<0.001***^†^	4.84	<0.001***^†^
**Lacunes (count)**				
Lobar	3.22	0.001**^†^	3.78	<0.001***^†^
Deep	−0.59	0.555	−1.68	0.086
Total number	3.03	0.002**^†^	−1.87	0.371
Total SVD burden score	3.11	0.003**^†^	0.56	0.581

Model 1 adjusts for head motion only, Model 2 adjusts for head motion, age, gender, and diagnosis. Bold p-values signify statistical significance. *p < 0.05, **p < 0.01, ***p < 0.001. ^†^Statistically significant after correcting for multiple comparisons using Holm–Bonferroni correction.

### Relative Contribution of SVD Markers to PSMD

Dominance analysis demonstrated that WMH volume (RW = 0.19, DW = 0.14) outperformed EPVS (RW = 0.04, DW = 0.003), microbleeds (RW = 0.05, DW = 0.02), and lacunes (RW = 0.02, DW = 0.01) in predicting PSMD. EPVS and microbleeds were comparable in dominance, although both outperformed lacunes in predicting PSMD. The same findings were observed when WMH volume was replaced by the semi-quantitative measure of WMH using the Fazekas scale (RW = 0.14, DW = 0.10).

### Regional Contribution to PSMD

Within each SVD marker, we examine the relative contribution of regional burden to PSMD using dominance analysis and relative weight analysis. For WMH, dominance analysis showed that PVH was a stronger predictor of PSMD, compared to DWMH – this was true for both semi-quantitative (Fazekas) and quantitative (volume) measurements of WMH (Table 3). Using Fazekas ratings, PVH contributed 66.4%, while DWMH accounted for 33.6% of *R*^2^ in the model, and 71.7 and 28.3%, respectively, for volumetric WMH measurements. EPVS-BG and EPVS-CSO were comparable in their contribution to PSMD (60.3 and 38.8%, respectively), although both regions were more predictive than midbrain EPVS (0.9%). Microbleeds and lacunes in lobar regions (58.0 and 85.1%, respectively) contributed significantly more to the PSMD score, compared to the same lesions in deep brain regions (42.0 and 14.9%, respectively; Table 3).

**TABLE 3:** Relative weight analysis and dominance analysis comparing regions within each SVD marker.

	β	*p*	Relative weight	% contribution to *R*^2^	Dominance weight
**WMH (Fazekas)**					
Periventricular	0.339	< 0.001*	0.126	66.4	0.075^a^
Deep	0.140	0.135	0.064	33.6	0.013
**WMH (volume)**					
Periventricular	0.503	< 0.001*	0.193	71.7	0.117^a^
Deep	0.022	0.844	0.077	28.3	< 0.001
**EPVS**					
Centrum semiovale	0.179	0.038*	0.044	38.8	0.027^b^
Basal ganglia	0.243	0.005*	0.070	60.3	0.052^b^
Midbrain	–0.048	0.558	< 0.001	0.9	0.002
**Microbleeds**					
Lobar	0.225	0.107	0.053	58.0	0.017^c^
Deep	0.089	0.523	0.038	42.0	0.003
**Lacunes**					
Lobar	0.241	0.004*	0.054	85.1	0.057^d^
Deep	–0.114	0.168	0.009	14.9	0.013

*Significantly associated with PSMD (p < 0.05). ^a^In dominance analysis, periventricular WMH dominated deep WMH in contribution to PSMD. ^b^In dominance analysis, EPVS in the centrum semiovale and basal ganglia dominated midbrain EPVS in contribution to PSMD. ^c^In dominance analysis, lobar microbleeds dominated deep microbleeds in contribution to PSMD. ^d^In dominance analysis, lobar lacunes dominated deep lacunes in contribution to PSMD.

### Comparing the Contribution of SVD and Neurodegeneration to PSMD

To assess the relative contribution of cerebrovascular damage and general neurodegeneration in predicting PSMD score, we performed multiple linear regression analysis, relative weight analysis, and dominance analysis to comparing the relative importance of WMH volume, GM volume, and WM volume to PSMD. PSMD was associated with WMH volume (β = 0.492, *p* < 0.001), but not GM volume (β = −0.149, *p* = 0.181) or WM volume (β = −0.178, *p* = 0.111) in multiple regression analysis. Dominance analysis and relative weight analysis demonstrated that WMH burden contributed significantly more to PSMD score (RW = 0.25, DW = 0.24), compared to GM (RW = 0.06, DW = 0.01) and WM (RW = 0.06, DW = 0.11) volume. To account for different causes and severity of cognitive impairment, a second model was estimated whereby diagnostic group and ACE-R scores were further added into the equation. Similar findings were obtained from this second model, whereby WMH volume was the strongest contributor to PSMD (β = 4.03, *p* < 0.001).

### Region-Specific Associations

**FIGURE 2:**
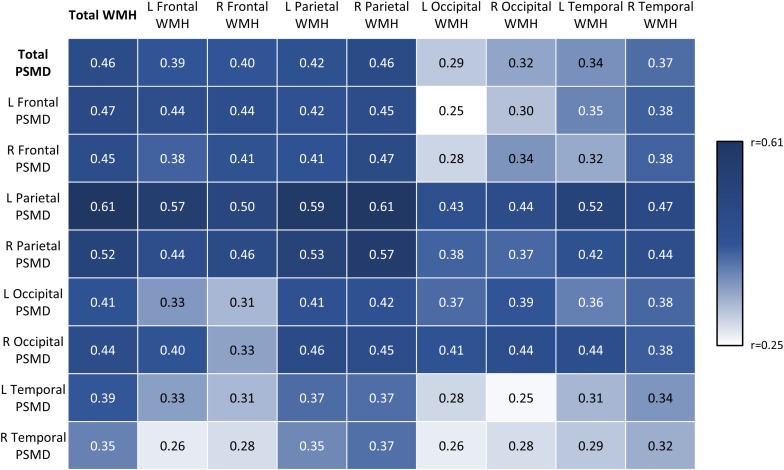
Robust correlation matrix depicting regional associations between PSMD and WMH volumes. Values represent robust correlation coefficients.

To examine whether regional PSMD scores corresponded with SVD burden in the same area, robust correlation analysis was conducted (Figure 2). All regional PSMD values were significantly associated with WMH volume across regions even after correcting for multiple comparisons using Holm–Bonferroni correction (*r* = 0.25–0.61, *p* ≤ 0.001–0.005) (Figure 2). Although significant, Fisher’s *r*-to-*z* transformations indicated that WMH was generally less correlated to temporal lobe PSMD (*r* = 0.25–0.39, *p* ≤ 0.001–0.004), while PSMD was generally less closely related to WMH in the occipital lobe (*r* = 0.25–0.44, *p* ≤ 0.001–0.005). Aside from these regions with weaker correlations, it was notable that associations were not significantly higher in corresponding areas (e.g., left frontal PSMD and left frontal WMH; *r* = 0.31–0.59, *p* < 0.001) compared to non-corresponding areas (e.g., left frontal PSMD and right temporal WMH; *r* = 0.25–0.61, *p* ≤ 0.001–0.005).

### PSMD and Cognition

We investigated associations between PSMD and global measures of cognition using robust linear regression analysis, adjusting for gender, age, and head motion. Higher PSMD was related to lower ACE-R (*t* = −4.07, *p* < 0.001) and MMSE scores (*t* = −2.13, *p* = 0.033). Dominance analysis indicated that PSMD contributed the most to ACE-R score (RW = 0.119, DW = 0.098), outperforming all SVD markers (RW = 0.004–0.035, DW ≤ 0.001–0.015) (Table 4).

**TABLE 4:** Dominance analysis to compare relative importance of PSMD and SVD markers in predicting cognition (ACE-R).

	β	*p*	Relative weight	% contribution to *R*^2^	Dominance weight
PSMD	**−0.373**	<0.001*	**0.119**	**68.1**	0.098*
WMH (volume)	−0.163	0.138	0.035	21.8	0.015
EPVS (basal ganglia)	0.164	0.083	0.011	3.5	<0.001
Microbleeds (count)	0.100	0.490	0.004	3.4	<0.001
Lacunes (count)	0.050	0.360	0.004	3.1	<0.001

PSMD (bold) dominated all other variables in predicting ACE-R score; *statistical significance at p < 0.001.

**FIGURE 3:**
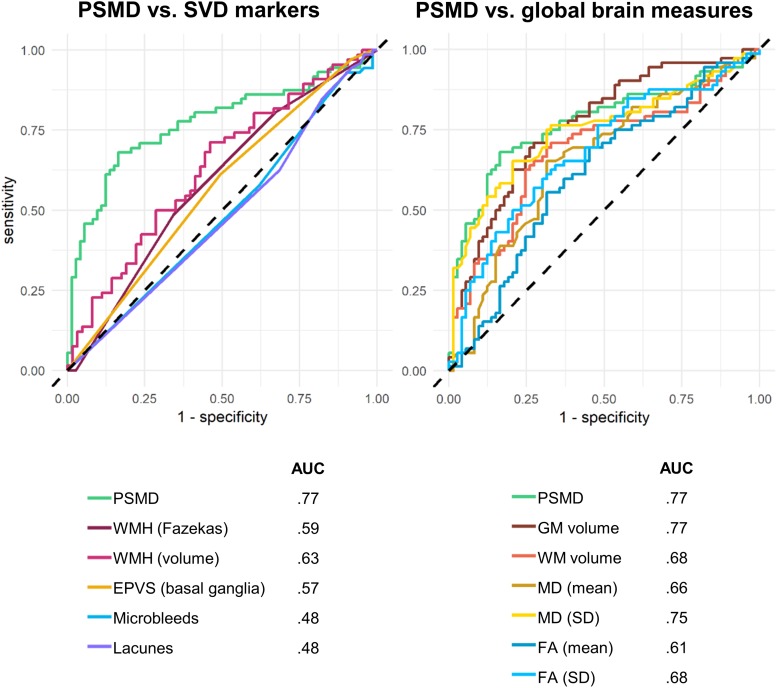
Receiver operating characteristics (ROC) curves separating cognitively impaired from cognitively healthy participants.

Receiver operating characteristic analysis was conducted to evaluate the ability of PSMD to discriminate between participants with and without cognitive impairment, as defined by an ACE-R cut-off of 83 (Figure 3). PSMD performed moderately well at detecting cognitive impairment (AUC = 77%, 95% CI = 69–85%). AUC derived from ROC curves were compared against PSMD using Delong’s test. AUC of PSMD was significantly greater than the curves of all SVD markers (AUC = 0.48–0.63), including volumetric measurements of WMH (*p* = 0.014), which derived the largest AUC (AUC = 63%, 95% CI = 54–73%) among all SVD markers. The AUCs of semi-quantitative SVD measures ranged from 48 to 59%, which were all significantly smaller than the PSMD (*p* < 0.01). The ROC curves of global brain measures were also assessed. Delong’s tests indicated that PSMD performed similarly to GM volume (*p* = 0.901), WM volume (*p* = 0.146), and MD standard deviation (*p* = 0.264) in detecting cognitive impairment, but outperformed FA measures (*p* = 0.010–0.025) and mean MD (*p* = 0.004) (Figure 3).

## Discussion

This study demonstrated robust associations between PSMD and the four core markers of SVD, both globally and regionally. PSMD appeared to be driven by these markers of vascular pathology, more so than markers of generalized neurodegeneration (GM and WM atrophy). Regional analysis showed that PSMD was associated with WMH across the entire brain, and that regional PSMD was not more closely related to WMH in corresponding regions, supporting the notion that PSMD is a measure of diffuse pathology. In comparison to traditional SVD markers, PSMD was more strongly associated with cognition, and performed better at detecting cognitive impairment, although it performed similarly to global brain measures.

Expanding on existing research, which has mostly examined PSMD in relation to WMH (Baykara et al., 2016; Deary et al., 2019), this present study demonstrated that associations with PSMD were not confined to WMH, but extended to other key SVD markers of EPVS, lacunes, and microbleeds, and total SVD burden. Nearly all global and regional measurements of SVD were associated with PSMD, with exception of deep lacunes and EPVS in the midbrain (Table 2). In terms of relative contribution, WMH was the strongest predictor of PSMD compared to other SVD markers.

Within each SVD marker, regional burden was compared for their relative contribution to PSMD. In terms of WMH, PSMD was more closely related to PVH than DMWH, a pattern characteristic of hypertensive arteriopathy. This suggests that PSMD may be more strongly affected by underlying vascular pathology affecting deep structures, rather than observable parenchymal alterations of DWMH. In line with DTI parameters in general, this demonstrates the ability of PSMD to detect microstructural changes in normal appearing WM that are not yet visible on conventional MRI (Van Norden et al., 2012). EPVS-CSO and EPVS-BG performed similarly well in predicting PSMD, although both contributed more to PSMD than EPVS-MB. Finally, focal cerebrovascular lesions in the form of lacunes and microbleeds were more strongly associated with PSMD when they occurred in lobar (as opposed to deep) regions. Taken together, PSMD appears to be sensitive to both (1) early microstructural tissue damage of vascular pathology, and (2) diffuse distribution of cerebrovascular lesions, positioning it as a valuable marker of overall SVD burden.

White matter hyperintensities outperformed global measures of brain volume as a predictor of PSMD, supporting earlier findings that PSMD was associated with SVD but not neurodegeneration (Baykara et al., 2016). The exclusivity of this relationship is an important feature that supports the use of PSMD as a valid and specific marker of SVD. Baykara et al. (2016) arrived at the same conclusion through their observation that PSMD was associated with WMH load, but did not differ between healthy controls, patients with mild cognitive impairment and low WMH, and Alzheimer’s disease patients with low WMH. Building upon this, we adopted a different approach by comparing WMH volume directly with volumetric measures of brain atrophy in relation to PSMD. Encouragingly, our findings supported the argument that PSMD was specific to vascular pathology, and not generalized neurodegeneration. However, given the heterogeneous etiologies of WMH, it is possible that associations between WMH and PSMD may reflect a combination of vascular and non-vascular pathologies (Habes et al., 2018; Alber et al., 2019).

Regional associations between PSMD and WMH supported the notion of PSMD as a diffuse marker of overall cerebrovascular burden. Results demonstrated that PSMD, regardless of location, was associated with WMH load across all regions. Importantly, associations between PSMD and WMH were not significantly stronger in corresponding regions (e.g., left frontal PSMD and left frontal WMH) compared to non-corresponding regions, supporting the PSMD’s position as a measure of diffuse pathology.

Finally, significant associations were found between PSMD and cognitive performance, independent of gender, age, diagnosis, and head motion during scanning. This was in line with existing PSMD research (Baykara et al., 2016; Deary et al., 2019; Vinciguerra et al., 2019; Wei et al., 2019). Importantly, PSMD was found to be a stronger predictor of cognitive performance than conventional markers of SVD. This was further supported by ROC analysis which demonstrated the PSMD’s ability to distinguish cognitively impaired from cognitively healthy participants with greater accuracy than traditional markers of SVD. The lack of association with SVD markers like lacunes and microbleeds may stem from a lack of statistical power, given the low incidence of such lesions. On the other hand, PSMD performed similarly to GM and WM volumes. This is perhaps unsurprising, given that majority of participants were patients with cognitive impairment/dementia wherein structural atrophy is a key characteristic. We also compared PSMD to conventional measures of DTI, to determine if positive PSMD findings were a simple reflection of the general superiority of DTI measures. We found that PSMD was better than mean FA and MD values at detecting cognitive impairment, although it was comparable to the standard deviation of MD in the brain. In contrast to global brain measures, PSMD may be able to detect cognitive deficits stemming from vascular pathology. Unfortunately, the cognitive tests used in this study were global measures of cognition, which are less sensitive to SVD-related changes compared to tests of executive function and information processing speed – using such measures may produce even stronger associations.

A key strength of this study was the inclusion of all four major MRI markers of SVD, and region-specific SVD measures. Furthermore, this study included both (1) *semi-quantitative* visual rating of WMH, which are more applicable to clinical settings, and (2) *quantitative* volumetric measurements of WMH, which are more sensitive and reliable at detecting WM changes. However, this study was limited by a number of factors. Firstly, the study sample comprised a heterogeneous mix of dementia diagnoses. This heterogeneity may reduce power to detect differences and emphasizes commonalities in the associations across diagnostic groups. However, it should not prevent investigations on the sensitivity of PSMD and its relationship to other measures. Furthermore, this transdiagnostic approach allowed us to determine whether results were independent of diagnostic grouping, which we were able to demonstrate through the consistency of results even after inclusion of diagnostic group as a covariate in regression models.

Future research should examine the sensitivity of PSMD to longitudinal SVD progression, investigate PSMD in relation to other known correlates of SVD such as vascular risk factors (e.g., hypertension) and other brain imaging measures (e.g., cerebral hypoperfusion, blood–brain barrier permeability), and assess its association to specific cognitive domains (e.g., executive function, processing speed). However, given the sensitivity of PSMD to head motion during scans, this should be controlled for in all intended use of the PSMD score.

## Conclusion

This present study demonstrates the PSMD’s sensitivity to vascular pathology instead of just non-specific neurodegeneration, and PSMD’s ability to detect microstructural vascular damage that is not visible on conventional MRI. Furthermore, PSMD appeared to be a diffuse marker of overall SVD burden, capturing different types of cerebrovascular alterations across the whole brain. This marker was also clinically relevant in terms of detecting cognitive impairment, and surpassed conventional MRI markers of SVD and DTI measures. Given the convenience of its fully automated, publicly available computation method, the PSMD is well-positioned as a promising new marker of SVD which can be easily implemented in large samples.

## Data Availability Statement

The datasets generated for this study are available on request to the senior author, for non-commercial academic studies, but may be subject to some restrictions according to consent and confidentiality.

## Ethics Statement

The studies involving human participants were reviewed and approved by the Cambridge Central Research Ethics Committee (reference: 13/EE/0104). The patients/participants provided their written informed consent to participate in this study.

## Author Contributions

AL developed the research question, visually rated SVD markers, performed the imaging analysis and quality checks on processed imaging data, conducted the statistical analysis, and wrote and manuscript. EM reviewed the data and manuscript, and the drafts, and provided the critical feedback. MM conducted the data collection, database management, reviewed the drafts, and provided the critical feedback. NN performed quality checks on processed imaging data, reviewed the drafts, and provided the critical feedback. JS was the second rater of SVD markers, reviewed the drafts, and provided the critical feedback. GS and LC were involved in data collection and reviewed the drafts. HM provided the critical feedback and made revisions to the manuscript. JO’B and JR reviewed the data and manuscript, provided the critical feedback, made revisions to the manuscript, designed the study protocols, supervised the study, and are the co-principal investigators of the study.

## Conflict of Interest

The authors declare that the research was conducted in the absence of any commercial or financial relationships that could be construed as a potential conflict of interest.

## Footnotes

1 https://www.joindementiaresearch.nihr.ac.uk

2 http://www.psmd-marker.com

3 http://fsl.fmrib.ox.ac.uk/fsl

4 http://www.fil.ion.ucl.ac.uk/spm/
